# Expanding the Clinical Spectrum Associated With *GLIS3* Mutations

**DOI:** 10.1210/jc.2015-1827

**Published:** 2015-08-10

**Authors:** P. Dimitri, A. M. Habeb, F. Garbuz, A. Millward, S. Wallis, K. Moussa, T. Akcay, D. Taha, J. Hogue, A. Slavotinek, J. K. H. Wales, A. Shetty, D. Hawkes, A. T. Hattersley, S. Ellard, E. De Franco

**Affiliations:** Department of Paediatric Endocrinology (P.D.), Sheffield Children's NHS Foundation Trust, Sheffield S10 2TH, United Kingdom; Paediatric Department (A.M.H.), Prince Mohamed Bin Abdulaziz Hospital, National Guard Health Authority, Al-Madinah, Riyadh 14214, Kingdom of Saudi Arabia; Ankara Pediatric Hematology Oncology Education and Training Hospital (F.G.), Ankara, Turkey; Diabetes Clinical Research Centre (A.M.), Plymouth Hospitals NHS Trust, Derriford PL6 8DH, United Kingdom; Department of Paediatrics (S.W.), Bradford Teaching Hospitals NHS Foundation Trust, Bradford, West Yorkshire BD9 6RJ, United Kingdom; Paediatric Department (K.M.), Maternity and Children Hospital, Jeddah 23342, Kingdom of Saudi Arabia; Kanuni Sultan Süleyman Education and Research Hospital (T.A.), 34303 Küçükçekmece, Istanbul, Turkey; Division of Pediatric Endocrinology (D.T.), Children's Hospital of Michigan, Wayne State University, Detroit, Michigan 48201; Department of Paediatrics (J.J.), Madigan Army Medical Center, Tacoma, Washington 98431; Institute for Human Genetics (A.S.), University of California, San Francisco, California 94143; Department of Paediatric Endocrinology and Diabetes (J.K.H.W.), Lady Cilento Children's Hospital, South Brisbane, Queensland 4101, Australia; Department of Paediatrics (A.S.), Nevill Hall Hospital, Abergavenny NP7 7EG, Wales, United Kingdom; Department of Paediatrics (D.H.), Royal Gwent Hospital, Newport NP20 2UB Wales, United Kingdom; and Institute of Biomedical and Clinical Science (A.T.H., S.E., E.D.F.), University of Exeter Medical School, EX2 5DW, United Kingdom

## Abstract

**Context:**

*GLIS3* (GLI-similar 3) is a member of the GLI-similar zinc finger protein family encoding for a nuclear protein with 5 C_2_H_2_-type zinc finger domains. The protein is expressed early in embryogenesis and plays a critical role as both a repressor and activator of transcription. Human GLIS3 mutations are extremely rare.

**Objective:**

The purpose of this article was determine the phenotypic presentation of 12 patients with a variety of *GLIS3* mutations.

**Methods:**

*GLIS3* gene mutations were sought by PCR amplification and sequence analysis of exons 1 to 11. Clinical information was provided by the referring clinicians and subsequently using a questionnaire circulated to gain further information.

**Results:**

We report the first case of a patient with a compound heterozygous mutation in *GLIS3* who did not present with congenital hypothyroidism. All patients presented with neonatal diabetes with a range of insulin sensitivities. Thyroid disease varied among patients. Hepatic and renal disease was common with liver dysfunction ranging from hepatitis to cirrhosis; cystic dysplasia was the most common renal manifestation. We describe new presenting features in patients with GLIS3 mutations, including craniosynostosis, hiatus hernia, atrial septal defect, splenic cyst, and choanal atresia and confirm further cases with sensorineural deafness and exocrine pancreatic insufficiency.

**Conclusion:**

We report new findings within the *GLIS3* phenotype, further extending the spectrum of abnormalities associated with *GLIS3* mutations and providing novel insights into the role of *GLIS3* in human physiological development. All but 2 of the patients within our cohort are still alive, and we describe the first patient to live to adulthood with a *GLIS3* mutation, suggesting that even patients with a severe *GLIS3* phenotype may have a longer life expectancy than originally described.

Permanent neonatal diabetes (PND) and congenital hypothyroidism can result from a number of genetic mutations. Mutations in *KCNJ11* and *ABCC8* genes, encoding the Kir6.2 and SUR1 subunits of the pancreatic ATP-sensitive potassium (K_ATP_) channel involved in regulation of insulin secretion, account for about half of cases of PND (1). Mutations in the *INS* gene leading to the disruption of insulin synthesis also result in PND (2). Further possible candidate genes for PND include *GCK*, *PDX1*, *GATA6*, *NEUROD1*, *NEUROG3*, *NKX2-2*, *IER3IP*, *PTF1A*, *HNF1B*, *RFX6*, and *MNX*. Syndromes that incorporate PND include immunodysregulation polyendocrinopathy enteropathy X-linked syndrome (*FOXP3*), Wolcott-Rallison syndrome (*EIF2AK3*), and pancreatic agenesis (*PDX1*, *PTF1A*, *GATA6*, and *GATA4*) (1, 3). Mutations in *TSHR*, *PAX8*, *NKX2-1*, *FOXE1*, and *NKX2-5* lead to congenital structural thyroid abnormalities, and thyroid dyshormonogenesis derives from mutations in *DUOX2*, *SLC5A5*, *TG*, *TPO*, and *DEHAL1* (4). Mutations in *GLIS3* (Gli-similar 3) result in the concomitant presentation of PND and congenital hypothyroidism. *GLIS3*, a member of the GLI-similar zinc finger protein family encoding for a nuclear protein with 5 C_2_H_2_-type zinc finger domains, maps to chromosome 9p24.3-p23 (OMIM 610192) (5). The protein is expressed early in embryogenesis and plays a critical role as both a repressor and activator of transcription (5, 6). It is specifically involved in the development of pancreatic β-cells, the thyroid, eye, liver, and kidney although tissue expression occurs to a lesser extent in the heart, skeletal muscle, stomach, brain, adrenal gland, and bone (7, 8). In 2003, Taha et al (9) described a consanguineous Saudi Arabian family in which 2 of 4 siblings had PND associated with intrauterine growth retardation (IUGR), congenital hypothyroidism, facial anomalies, congenital glaucoma, hepatic fibrosis, and polycystic kidneys, described as neonatal diabetes and hypothyroidism (NDH) syndrome (9). A third child from that family consequently died of the same condition (10). Genome-wide linkage analysis and sequencing of candidate genes performed on this family by Senee et al (8) in 2006 identified a homozygous frameshift mutation (c.1873dupC, previously reported as 2067insC) in the *GLIS3* gene, which is likely to result in transcript degradation by nonsense mediated decay (6). Both children with this mutation died in infancy. Senee et al (8) described 2 further families with mutations in *GLIS3*. The first harbored a homozygous 426-kb deletion, which encompassed the *SLC1A1* gene and part of *GLIS3.* The affected offspring in the other family carried a homozygous 149-kb deletion that included a portion of *GLIS3* as well; the region common to both deletions mapped to the known start codon of *GLIS3.* Patients in these 2 families presented a milder phenotype. Variations in the GLIS3 phenotype have been attributed to the tissue-specific expression of variable-length transcripts derived from the 11-exon *GLIS3* gene. The absence of pancreatic and thyroid GLIS3 transcripts in the 2 families with deletions resulted in neonatal diabetes and hypothyroidism and the absence of an eye-specific transcript in 1 family resulted in congenital glaucoma. The absence of renal and hepatic abnormalities was attributed to the unaltered expression of liver- and kidney-specific transcripts. More recently, an extended phenotype associated with mutations in *GLIS3* has been reported, including skeletal abnormalities and exocrine pancreatic dysfunction (11). Given the rarity of this condition, further information relating genotype to phenotypic manifestation is required.

We describe a case series of 12 patients with mutations in *GLIS3*, providing additional insight into the clinical features associated with this rare condition.

## Subjects and Methods

The study was conducted in accordance with the Declaration of Helsinki principles with informed parental consent given on behalf of children. Clinical information was provided by the referring clinicians via a neonatal diabetes request form (available at www.diabetesgenes.org), from clinical notes, and subsequently by using a questionnaire circulated to referring clinicians to gain further information.

### Genetic analysis

*GLIS3* gene mutations were sought by PCR amplification (primer sequences are available on request) and sequence analysis of exons 1 to 11 by comparison with the reference sequence NM_001042413. Exon 1 is noncoding (the 5′ untranslated), and the start codon is located within exon 2.

The effect of coding variants on the protein was investigated in silico using the bioinformatic tool Alamut (Interactive Biosoftware). When PCR amplification failed, suggesting a homozygous deletion, parental samples were investigated by real-time quantitative PCR on an ABI 7900 system (TaqMan assay with SYBR Green detection), and the copy number of exons 1 to 11 was determined by the 2^−ΔΔ^*C_t_* method.

Patients 1 and 10 were analyzed for all of the known neonatal diabetes genes using a targeted next-generation assay (12). Mutations identified by this assay were confirmed by Sanger sequencing.

## Results

**Figure 1.**
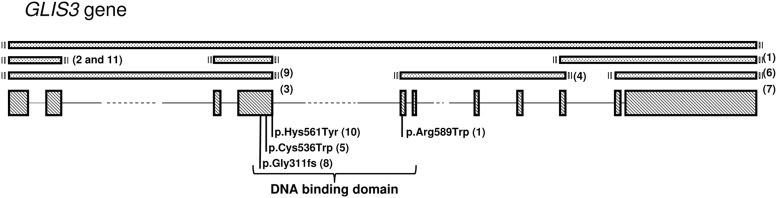
Schematic representation of the *GLIS3* gene. The diagonal boxes represent the exons. The bracket indicates the region encoding the zinc-finger DNA binding domain. Mutation positions are indicated under the gene. Deletions are represented as dotted boxes. The patient number for each mutation/deletion is indicated in parentheses.

Table 1 describes the nucleotide and predicted protein changes of the *GLIS3* mutations identified in our case series. Deletions of ≥1 of the 11 exons of *GLIS3* were observed in most patients. Patients 1, 5, and 10 harbor missense mutations (p.Arg589Trp, p.Cys536Trp, and p.His561Tyr, respectively), affecting highly conserved amino acids located in the DNA binding domain and so are likely to be pathogenic, thus severely affecting the function of the GLIS3 protein. Patient 1 is the first patient reported to be a compound heterozygote for 2 mutations in *GLIS3* (a deletion and a missense mutation). Patients 3a and 3b are siblings. Figure 1 provides a schematic representation of *GLIS3*, showing the mutations described in our patient population.

**Table 1.** Mutations and Nucleotide Changes Relating to Mutations in GLIS3

Patient No.	Exon	Mutation	Nucleotide Change	Previously Published	In Silico Prediction
1	5	p.Arg589Trp/exons 1–11 del	c.1765C>T/c.-?_2793+?del	No	Pathogenic/pathogenic
2	1–2	Exons 1–2 del/exons 1–2 del	c.-?_388+?del/c.-?_388+?del	Yes^7^	Pathogenic
3a	1–4	Exons 1–4 del/exons 1–4 del	c.-?_1710+?del/c.-?_1710+?del	Yes^7^	Pathogenic
3b	1–4	Exons 1–4 del/exons 1–4 del	c.-?_1710+?del/c.-?_1710+?del	No	Pathogenic
4	5–9	Exons 5–9 del/exons 5–9 del	c.1711-?_2473+?del/c.1711-?_2473+?del	Yes^6^	Pathogenic
5	4	p.Cys536Trp/Cys536Trp	c.1608C>G/c.1608C>G	Yes^6^	Pathogenic
6	9–11	Exons 9–11 del/exons 9–11 del	c.2298-?_2657+?del/c.2298-?_2657+?del	No	Pathogenic
7	10–11	Exons 10–11 del/exons 10–11 del	c.2474-?_2793+?del/c.2474-?_2793+?del	No	Pathogenic
8	4	p.Gly311Alafs/p.Gly311Alafs	c.932delG/c.932delG	No	Pathogenic
9	3–4	Exons 3–4 del/exons 3–4 del	c.389-?_c.1710+?del/c.389-?_c.1710+?del	No	Pathogenic
10	4	p.His561Tyr/p.His561Tyr	c.1681C>T/c.1681C>T	No	Pathogenic
11	1–2	Exons 1–2 del/exons 1–2 del	c.-?_388+?del/c.-?_388+?del	No	Pathogenic

The clinical features of all of the patients are summarized in Table 2. All but 2 of the patients in our case series remain alive to date, and patient 1 is the first patient with a *GLIS3* mutation to survive into adulthood (aged 36 years). Patient 6 died from liver failure with marked portal hypertension and esophageal variceal bleeding, and patient 9 died of overwhelming measles sepsis and multiorgan failure at 6 months of age. Nine patients in our cohort were born to parents who were first cousins. Patient 3, who was born to apparently not related parents was described previously by Dimitri et al (11). In this case series, we describe another child with a *GLIS3* mutation born to Caucasian parents who are not related (patient 1) and the sibling of patient 3a (patient 3b). A homozygous deletion was confirmed in patient 6; however, consanguinity was denied by the patient's parents.

**Table 2.** Clinical Features Presenting in Patients With GLIS3 Mutations

Patient No.	Exon	Birth Weight, g	IUGR^a^	Gestation, wk	Ethnicity	Sex	Consanguineous	Age of Onset of PND^b^	Congenital Hypothyroidism	Liver Disease	Kidney Disease	Exocrine Pancreatic Disease	Congenital Glaucoma	Skeletal Disease	Developmental Delay	Facial Dysmorphism	Other Features	Alive	Current Age, y
1	5	2750	No	39	Caucasian	Female	No	30 h	No	No	No	No	No	No	Yes	No	Choanal atresia, hiatus hernia	Yes	36 y
2	1–2	1170	Yes	35	Bangladeshi	Female	Yes	3 d	Yes	Yes	Yes	Yes	No	Yes	Yes	Yes	No	Yes	6.3 y
3a	1–4	1430	Yes	35	Caucasian	Male	No	4 d	Yes	Yes	Yes	Yes	No	No	Yes	Yes	Bilateral sensorineural deafness, PDA, pancreatic cyst	Yes	6.02 y
3b	1–4	2020	Yes	38	Caucasian	Male	No	2 d	Yes	Yes	Yes	Yes	No	No	Yes	Yes	Pancreatic cysts, splenic cyst, bilateral sensorineural deafness	Yes	20 mo
4	5–9	1750	Yes	34	Arab	Female	Yes	2 d	Yes	Yes	Yes	No	No	No	Yes	Yes	Yes	Yes	4.7 y
5	4	2050	Yes	39	Arab	Male	Yes	5 d	Yes	No	No	No	No	Yes	Yes	No	No	Yes	6.8 y
6	9–11	1530	Yes	37	African-American	Female	Unknown	7 d	Yes	Yes	Yes	No	Yes	Yes	Yes	Yes	No	No	6.0 y
7	10–11	1235	Yes	36	Yemeni	Female	Yes	3 d	Yes	Yes	Yes	Yes	Yes	No	Yes	Yes	No	Yes	3 y
8	4	1860	Yes	39	Pakistani	Female	Yes	24 h	Yes	No	Yes	No	No	Yes	No	No	Right sensorineural deafness	Yes	2.5 y
9	3–4	1520	No	30	Turkish	Male	Yes	21 d	Yes	Yes	Yes	No	No	No	Yes	Yes	No	No, died at 6 mo of age	NA
10	4	973	Yes	31	Kurdish	Male	Yes	31 d	Yes	Yes	Yes	No	Yes	No	No	Yes	Patent ductus arteriosus	Yes	4.5 y
11	1–2	1730	Yes	39	Arab	Male	Yes	19 d	Yes	No	Yes	No	Yes	No	No	Yes	Ostium secundum ASD	Yes	7 mo

Abbreviations: ASD, atrial septal defect; NA, not applicable; PDA, patent ductus arteriosus.

a Birth weight <10th centile for gestational age.

b Permanent neonatal diabetes.

PND was the only consistent feature of all of our patients with *GLIS3* mutations. Age at diagnosis ranged from birth to 23 days. All patients were insulin treated. Patients were initially treated with insulin at 0.4 to 2.0 U/kg/24 h (patient 4 required 2.0 U/kg/24 h), demonstrating a range of insulin sensitivities. Patient 11 had high insulin sensitivity, leading to recurrent hypoglycemic episodes with very small doses of insulin. Others had labile blood glucose (patients 3a and 3b), and one patient clinically demonstrates insulin resistance, particularly during periods of illness (patient 2). In the first year of life, this patient required 0.5 to 0.7 U/kg of insulin per day. However, during times of intercurrent illness, doses of insulin at 3 to 4 times her normal requirement were required to achieve normoglycemia. Despite erratic blood glucose control with periods of insulin resistance, her glycosylated hemoglobin at 1 year of age was 7.8% (62.0 mmol/mol).

Apart from patient 1, all patients had with congenital hypothyroidism. This is a cardinal feature of the NDH syndrome described previously in all patients with *GLIS3* mutations(8, 9). Although congenital hypothyroidism presented during the first week of life in all patients, the patterns of thyroid disease were variable. Patients 2, 3a, and 3b had elevated TSH levels that were resistant to treatment with thyroxine as described previously (11). Similarly, patients 7 and 11 had very high TSH levels that did not reduce to normal limits with levothyroxine therapy despite normalizing of free T_4_. In these 3 patients, the thyroid anatomy was normal on ultrasonography. In contrast, patient 4 presented with congenital hypothyroidism due to athyreosis and responded to levothyroxine at a starting dose of 28 μg/kg/d. In patients 5 and 8, thyroid ultrasonography was not performed. However, their initial levothyroxine requirements were 24 and 15 μg/kg/d, respectively. Patient 8 had fluctuating levels of TSH (20–30 mIU/L) in the first year of life despite normal levels of free T_4_, which have since normalized. Patients 6 and 10 had normal thyroid anatomy on ultrasonography, and in comparison with other patients with *GLIS3* mutations, TSH responded appropriately to conventional doses of levothyroxine with an appropriate increase in the levothyroxine dose with age. Notably, the postmortem examination of the thyroid gland in patient 6 demonstrated a paucity of colloid as well as extensive perifollicular and interstitial fibrosis, explaining the need for thyroxine despite apparently normal thyroid anatomy on ultrasonography.

**Figure 2.**
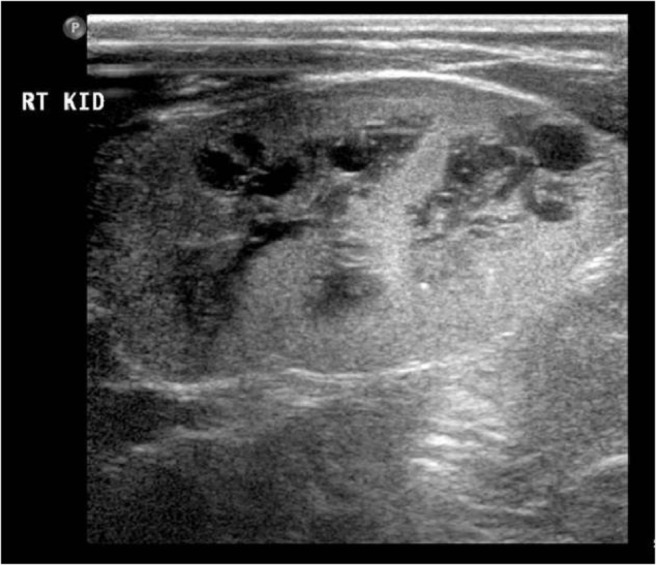
Extensive renal cystic dysplasia in patient 2 with a mutation in *GLIS3* at 5 months of age.

Liver disease was documented in 7 of our 12 patients. The hepatic dysfunction presented concomitantly with renal abnormalities and ranged from hepatitis (patients 3b and 4) to hepatic fibrosis and cirrhosis (patients 2, 3a, 6, 7, 9, and 10). Nine patients have anatomical kidney changes. Of these, 7 children showed variable renal cystic dysplasia ranging from an isolated cyst observed in patient 10 and bilateral calyceal calcification in patient 11 to extensive cystic renal dysplasia (patients 2, 3a, 3b, 4, and 9) (Figure 2). Patient 7 lacks renal corticomedullary differentiation, and renal ultrasonography in patient 6 demonstrated bilateral renal enlargement with no cystic changes.

**Figure 3.**
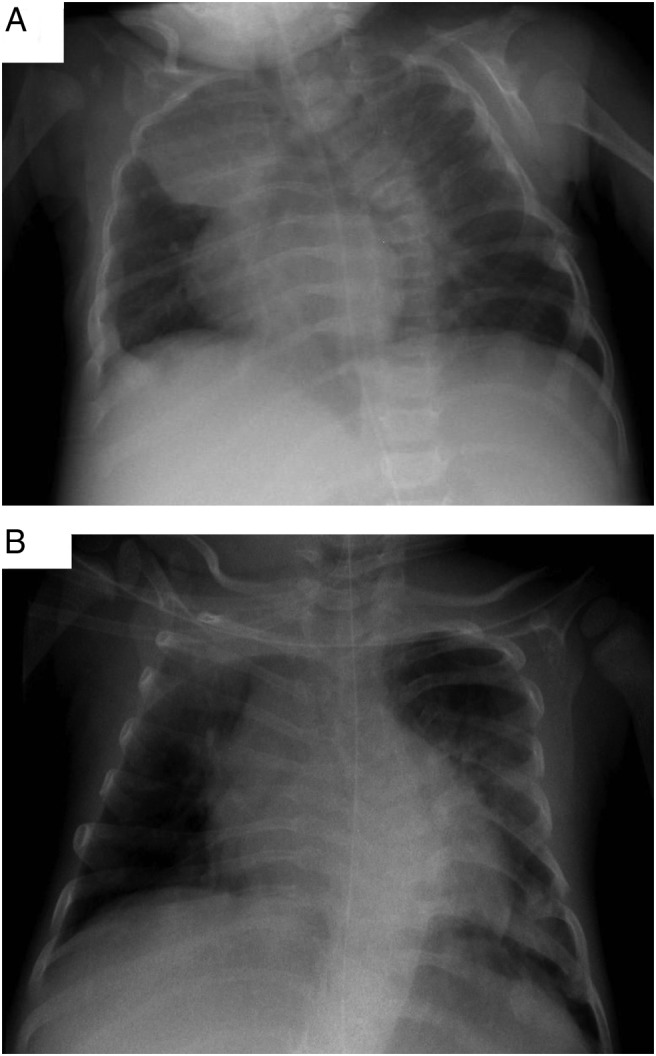
A, Anteroposterior chest x-ray of patient 2 performed on day 101 demonstrating thoracolumbar scoliosis and left rib fractures located on ribs 7, 8, and 9. B, Anteroposterior chest x-rays performed on day 412 demonstrating worsening thoracolumbar scoliosis and persistence of callus formation in left ribs 7, 8 and 9 with a new fracture at left rib 6.

Patient 2 was the first patient to be described with skeletal manifestations due to a mutation in *GLIS3*. She presented with osteopenia, significantly delayed rib fracture healing, and a marked thoracolumbar scoliosis (Figure 3, A and B). At 4 months of age, the PTH level was 62.6 ng/L (11–35 ng/L) with a 25-hydroxyvitamin D level of 34.6 nmol/L (50–90 nmol/L). Serum calcium and phosphate and bone alkaline phosphatase concentrations measured 2.56 mmol/L (2.13–2.72 mmol/L), 2.27 mmol/L (1.10–2.40 mmol/L), and 297.3 mmol/L (9–28 mmol/L), respectively. Despite normalization of the PTH and vitamin D levels after treatment with ergocalciferol and calcium, the patient sustained a further rib fracture on the left side (11). Patient 5 was also reported to have skeletal abnormalities with prominent right sixth and seventh ribs but normal bone biochemistry. Patient 6 is the first patient with a mutation in *GLIS3* to have sagittal craniosynostosis requiring surgical intervention. This patient initially manifested with mild hypocalcemia in the second month of life (7.8 mg/dL; normal range, 8.7–10.1 mg/dL), which subsequently normalized without treatment. Patient 8 was osteopenic by 6 months and despite vitamin D supplementation, her 25-hydroxyvitamin D_3_ level was 33.5 nmol/L at 6 weeks of age. Adjusted serum calcium in this patient was 2.0 mmol/L in the first week of life and subsequently normalized to 2.5 mmol/L by 12 days of age without calcium replacement. Alkaline phosphatase peaked at 3 months to 2100 U/L but had normalized by 2 years of age. No fractures were identified in patient 8.

Malabsorption due to exocrine pancreatic insufficiency as demonstrated by low fecal elastase was a feature in patients 2, 3a, 3b, and 7 in our case series. These 4 patients have been treated with pancreatic enzyme supplementation. We report the first patient with a *GLIS3* mutation to present with a splenic cyst (patient 3b).

Developmental delay and IUGR were common features in our patient cohort. Only 4 of 12 of our patients had congenital glaucoma although there was no clear relationship between the ocular presentation and the *GLIS3* exon affected as suggested by Senee et al (8).

Congenital cardiac defects in patients with *GLIS3* mutations have not been described previously. Patient 11 had an ostium secundum defect. It is likely that the patent ductus arteriosus observed in patients 3a and 10 was due to prematurity rather than to an abnormality in *GLIS3* function. Patient 1 is the first patient with a *GLIS3* mutation to present with choanal atresia. Before this study, only 1 patient had been described with sensorineural deafness (patient 3a) and a deletion of exons 1 to 4 of *GLIS3* (11). Patient 3b (sibling of patient 3a) also presented with bilateral sensorineural deafness; we now report a second patient of Pakistani origin (patient 8) with a frameshift mutation in exon 4 of *GLIS3* who had sensorineural deafness. Of the patients who presented with dysmorphic features, consistent features included low-set ears, epicanthic folds, a flat nasal bridge, and a long philtrum with a thin upper lip.

## Discussion

The variability in phenotype observed between our patients and others (8) has been attributed to the differential expression of multiple *GLIS3* transcripts. Two major transcripts, 7.5 kb and smaller (0.8–2.0 kb), have been described previously; the 7.5-kb transcript is strongly expressed in pancreas, thyroid, and kidney with smaller transcripts predominantly expressed in liver, kidney, heart, and skeletal muscle (8). Thus, mutations in *GLIS3* have the potential to cause widespread disruption. Previously described severely affected individuals with mutations in *GLIS3* appear to have total loss of function of the gene (8). For severely affected patients with deletions, it is likely that the mutated transcripts undergo nonsense-mediated decay and little or no protein is produced. Patient 1 is the first patient described with biallelic *GLIS3* mutations, who does not have congenital hypothyroidism, and she has a milder phenotype than other patients in the cohort. She is a compound heterozygote for a whole-gene deletion and a missense mutation in exon 5 (p.Arg589Trp). We thus speculate that the missense mutation may be a hypomorphic change resulting in a mutated protein possessing some residual function.

The cardinal feature in all our patients was the diagnosis of neonatal diabetes in the first weeks of life, and the concomitant presence of IUGR may reflect significant intrauterine insulin deficiency. *GLIS3* plays a key role in pancreatic development, particularly in the embryogenesis of β-cells, which explains why our patients and others with *GLIS3* mutations to date have presented with PND (11). *GLIS3* interacts with key regulatory genes in pancreatic embryogenesis including *ONECUT1* and *NEUROGENIN3* (*NEUROG3*) (13–15). *GLIS3* expression also persists beyond the embryonic period, promoting β-cell proliferation and regulating insulin gene expression through binding to GLI-RE on the *INS* gene (16). Therefore, the variation in insulin sensitivities among patients (and mutations) may relate to the impact of the mutation on the nuclear localization, GLI-binding element activity, transactivation, pancreatic development, subsequent β-cell proliferation, and remnant endogenous insulin production. In humans, *GLIS3* has been identified as a susceptibility locus for the risk of type 1 and 2 diabetes (17, 18). The significant insulin requirements observed during periods of illness in our more severely affected patients may also suggest a possible role of GLIS3 on the end-organ response to insulin, possibly at the level of the insulin receptor. However, a large difference in insulin sensitivities was observed in patients with the same mutation in our cohort (patients 2 and 11). This finding suggests that other genetic factors might influence the insulin response in these patients. Initially, the abnormalities in the pancreas in patients with *GLIS3* mutations were thought to be limited to β-cells. However, *GLIS3* transcripts are highly expressed not only in pancreatic β-cells but also to a lesser degree in pancreatic acini. The presence of exocrine pancreatic dysfunction in patients 2, 3a, 3b, and 7 in our series suggests that exocrine pancreatic involvement may be more extensive than previously described (8). The presence of pancreatic cystic changes in patients 3a and 3b supports the previous observations that GLIS3 is important in the development and maintenance of pancreatic ducts (15).

The spectrum of structural thyroid abnormalities including athyreosis, glandular hypoplasia, and normal thyroid anatomy with lack of normalization of TSH after therapy with thyroxine in infancy demonstrates a broad range of thyroid dysfunction resulting from mutations within the same gene. A recent postmortem examination in patient 6 demonstrated a paucity of colloid as well as extensive perifollicular and interstitial fibrosis despite initially normal thyroid ultrasonography. No explanation for this variation has been offered to date although a similar phenotypic variability is also observed in patients with mutations in *PAX8* and *NKX2-1* (TTF1/NK2 homeobox-1 or thyroid transcription factor 1), involved in thyroid cell differentiation and proliferation and subsequent expression of genes encoding for thyroglobulin, thyroid peroxidase, thyrotropin receptor, and the sodium-iodide symporter (19–21). However, despite these phenotypic similarities, there is no evidence for conserved GLI transcription binding sites in the *PAX8*, *NKX2-1*, or *TSHR* flanking gene sequences. Further in vitro work is required to determine whether *GLIS3* works upstream of genes in pathways regulating thyroid development, hormonogenesis, and the end organ response to T4. The failure of suppression of TSH after thyroxine supplementation in some patients suggests an additional thyroid hormone resistance. Further work is required to understand the role of GLIS3 in thyroid hormone activity.

As described previously in patients with *GLIS3* mutations (8), most of our patients presented renal parenchymal disease, primarily renal cystic dysplasia. However, some patients with mutations in *GLIS3* did not develop renal disease. Whereas the variation in renal manifestations may be mutation related, out of 3 related patients with a homozygous insertion (2067insC) leading to a frameshift and likely degradation of the transcript reported by Senee et al (8), only 2 had renal cystic dysplasia. Similarly, the manifestations of hepatic disease varied among patients. *GLIS3* contains 29 known putative transcription start sites across the 11-exon gene, resulting in variable length transcripts. Larger (7.5 kb) and smaller (0.8–2.0 kb) transcripts are expressed in the kidney and smaller (0.8–2.0 kb) transcripts are expressed in the liver (8). The variable presentation of hepatic and renal disease therefore may be related to the relative qualitative and quantitative expression of tissue transcripts and the encoded proteins in individual patients or alternatively to the variation in the expression of regulatory transcripts.

Most patients described to date with *GLIS3* mutations also present dysmorphic features (8). In our cohort, dysmorphic features were seen in 9 of the 12 patients. GLIS3 is expressed during embryonic face development, which may help in part to explain the dysmorphic facial features observed in these patients (5). Congenital glaucoma was observed in patients 6, 7, 10, and 11 in our cohort. In mouse models, *Glis3* is expressed in a dynamic pattern during eye development, initially in the dorsal optic vesicle and subsequently in the lens and the retina, which supports the presentation of glaucoma in our patients and previous patients with *GLIS3* mutations (5). However, from our cohort we were unable to ascribe a specific exon relating to the eye disease.

Skeletal manifestations were first described in 2011 (11) in a patient with a *GLIS3* mutation presenting multiple rib fractures with persistence of callus formation and scoliosis associated with a deletion in exons 1 to 2. The persistence of callus formation suggests a defect in bone remodeling either due to dysfunctional osteoblast signaling to osteoclasts or reduced osteoclastic bone reabsorption. Milder skeletal abnormalities (prominence of the left ribs) were observed in patient 5 (Table 1), who carries a missense mutation in *GLIS3*, and osteopenia in patient 7, who also has a missense mutation in exon 4. Patient 6 presented with craniosynostosis, which is a novel presentation in the *GLIS3* phenotype. Recent evidence suggests a role of *GLIS3* in osteoblast differentiation by the up-regulation of fibroblast growth factor 18 (FGF18) (7, 22). A reduction in or absence of FGF18 results in delayed bone mineralization due to diminished osteoblast terminal differentiation and proliferation. The expression of *GLIS3* and WW domain containing transcription regulator 1 (*WWTR1*) overlaps in the kidney, and mutations in both genes result in renal cystic dysplasia with a high glomerular cystic load (23, 24). Similarly, WWTR1 and GLIS3 have a stimulatory role in osteogenesis while inhibiting adipogenesis (7). *WWTR1* interacts with *GLIS3* to enhance its transcriptional activity by acting as a coactivator. The C terminus of *GLIS3* is fundamental for this action (24). Thus, *GLIS3* mutations that affect the C-terminal domain abolish the interaction between these genes, which may in part explain the concomitant renal and skeletal manifestations seen in human *GLIS3* mutations.

We previously reported the first patient with a *GLIS3* mutation to present with sensorineural deafness (patient 3a) (11). We now report this finding in the sibling of patient 3a (patient 3b) and in another unrelated child (patient 8) with a frameshift mutation at exon 4. This mutation will introduce a premature stop codon, and the subsequent transcript will be degraded in a similar way to deletions in GLIS3. Because this mutation functionally has an effect to similar to that of deletions, we are unable to associate the hearing defect with a specific exon although exon 4 is affected in all 3 patients. For patients severely affected by *GLIS3* mutations, developmental delay and learning difficulties are common features. *GLIS3* is known to be expressed in brain tissue during embryogenesis, but there is little evidence to date connecting GLIS3 with brain development. Only 1 study to date, using genome-wide association studies of cerebrospinal fluid tau levels to identify risk variants for Alzheimer disease, has identified *GLIS3* as a significant locus for the development of Alzheimer disease (25). Further work in this area is required to understand how GLIS3 may alter cerebral embryogenesis and maturation.

In one of our families (patient 2) and in a family reported by Senee et al (8), the deletion also encompassed the gene encoding the neuronal/epithelial high-affinity glutamate transporter *SLC1A1* (solute carrier family 1). SLC1A1 is principally expressed in neurons, kidney, and small intestine. Mutations in *SLC1A1* are thought to cause dicarboxylic aminoaciduria (26) and have been associated with psychiatric disorders including psychosis, obsessive compulsive disorder, and neuronal degeneration (27, 28).

We have presented an extended spectrum of clinical features in relation to patients with mutations in the *GLIS3* gene. Our current study has focused on the clinical manifestations of patients with these mutations, and further in vitro work is required to test the *GLIS3* missense variants functionally in biological models.

In summary, patients presenting with mutations in *GLIS3* characteristically present with neonatal diabetes with variable insulin sensitivity and congenital hypothyroidism due to a range of underlying causes. Although mutations in *GLIS3* are more common in consanguineous pedigrees, we report 2 patients from apparently unrelated parents with *GLIS3* mutations. We also report the first patient with compound heterozygous mutations in *GLIS3* with preservation of thyroid function, who is also the first patient reported with a *GLIS3* mutation to survive into adulthood. Hepatic and renal disease is common in the patients in our cohort, but the presentation is variable. We report new findings within the *GLIS3* phenotype including cardiac disease, hiatus hernia, sagittal craniosynostosis, splenic cystic change, and choanal atresia, further extending the spectrum of abnormalities associated with *GLIS3* mutations and providing novel insights into the role of *GLIS3* in human physiological development. We report further patients presenting with exocrine pancreatic insufficiency and sensorineural deafness. All but 2 of the patients in our cohort are still alive, suggesting that even patients with a severe *GLIS3* phenotype may have a longer life expectancy than originally described.
